# Website Review: How to Get the Best From Fission Yeast Genome Data

**DOI:** 10.1002/cfg.175

**Published:** 2002-06

**Authors:** Valerie Wood, Jürg Bähler

**Affiliations:** The Wellcome Trust Sanger Institute Hinxton, Cambridge CB10 1SA UK

## Abstract

Researchers are increasingly depending on various centralized resources to access the vast amount of information reported in the literature and generated by systematic sequencing and functional genomics projects. Biological databases have become everyday working tools for many researchers. This dependency goes both ways in that the databases require continuous feedback from the research community to maintain accurate, reliable, and upto- date information. The fission yeast *Schizosaccharomyces pombe* has recently been sequenced, setting the stage for the post-genome era of this popular model organism. Here, we provide an overview of relevant databases available, or being developed, together with a compilation of Internet resources containing useful information and tools for fission yeast.


**Comparative and Functional Genomics**
*Comp Funct Genom* 2002; **3**: 282–288.
Published online 17 May 2002 in Wiley InterScience (www.interscience.wiley.com). **DOI:** 10.1002/cfg.175

**Feature**

### Website Review: How to get the best from fission yeast genome data

Valerie Wood and Jürg Bähler*
*The Wellcome Trust Sanger Institute, Hinxton, Cambridge CB10 1SA, UK*

*Correspondence to:
The Wellcome Trust Sanger Institute, Hinxton, Cambridge CB10 1SA, UK.
E-mail: jurg@sanger.ac.uk

Received: 20 February 2002
Accepted: 22 April 2002

### Abstract

**Researchers are increasingly depending on various centralized resources to access the vast amount of information reported in the literature and generated by systematic sequencing and functional genomics projects. Biological databases have become everyday working tools for many researchers. This dependency goes both ways in that the databases require continuous feedback from the research community to maintain accurate, reliable, and up-to-date information. The fission yeast *Schizosaccharomyces pombe* has recently been sequenced, setting the stage for the post-genome era of this popular model organism. Here, we provide an overview of relevant databases available, or being developed, together with a compilation of Internet resources containing useful information and tools for fission yeast. Copyright © 2002 John Wiley & Sons, Ltd.**

**Keywords: *Schizosaccharomyces pombe*; Databases; genome sequence; Internet resources; functional genomics; gene ontology; GeneDB**

The *Schizosaccharomyces pombe* genome sequence and a preliminary analysis have recently been reported [15], together with several articles celebrating this achievement [11,16,14]. This landmark will further establish and expand the role of fission yeast as a major experimental model organism. It will also increase the need for organized and continuously updated data repositories to allow online access to biological information for fission yeast and related data in other organisms. This paper provides a guide to available databases and other Internet resources relevant to fission yeast. We hope that colleagues will find this compilation helpful, whether they work with fission yeast or wish to access data on this model organism for computational or comparative analyses. We also describe how researchers can contribute to the development and contents of these resources, which is essential to provide accurate and current information for the community. Figure 1 shows the dataflow between the major databases and resources described in the text.

### 1. Repositories of genome sequence and annotation

### 1.1. The *S. pombe* genome project

**http://www.sanger.ac.uk/Projects/S_pombe**
The *S. pombe* genome project home page at the Sanger Institute will continue to maintain links to the following resources and primary datasets available to download by ftp:

- Clone resources
- Primary EMBL submissions
- Annotated sequence contigs (can be viewed using Artemis, see section 4)
- Assembly data
- FASTA format protein database
- Gene ontology association tables (see section 1.4)
- Chromosome map images
- Curated budding yeast ortholog table

Copyright © 2002 John Wiley & Sons, Ltd.

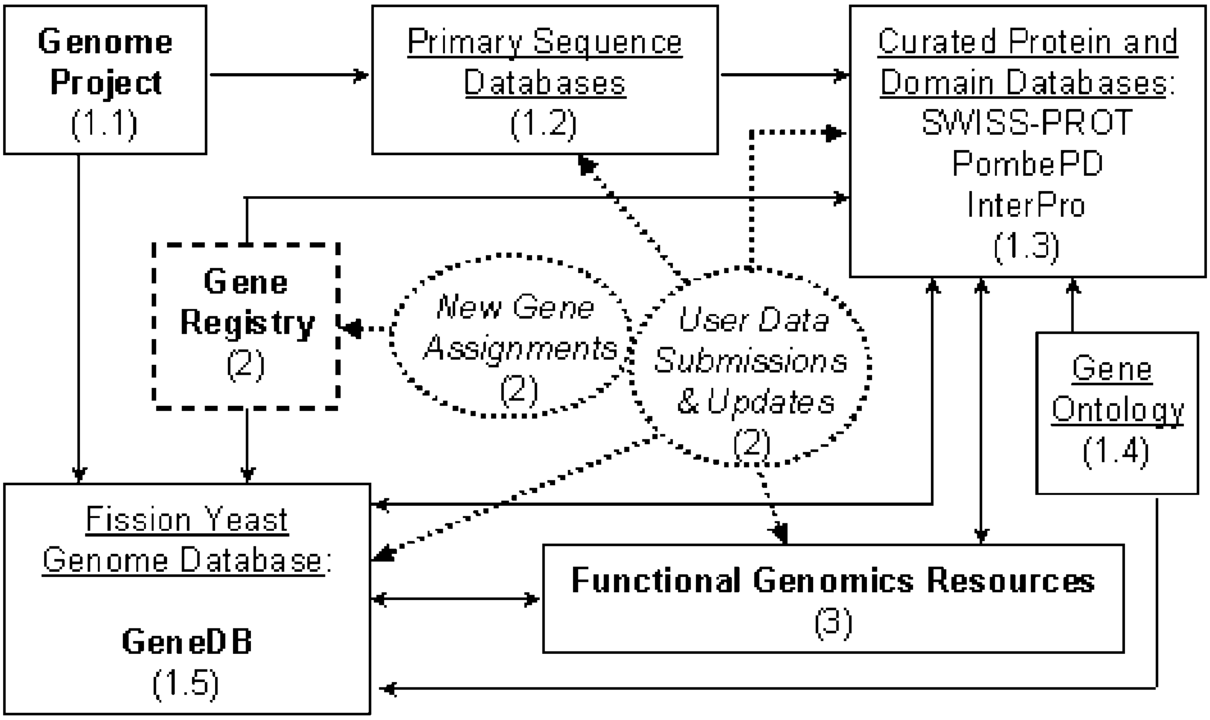

**Figure 1.** Scheme of genomic databases and resources and their relationship with each other. Chapter sections describing the various parts are given in parentheses. Arrows show the dataflow between the various databases and resources. Constant updates, new submissions, and feedback from specialized users are crucial to maintain accurate and up-to-date information in the databases (dotted arrows). Fission yeast specific tools are shown in **bold**, most of which are at an early stage of their development. Databases to pool functional genomic information from various organisms are also being developed and will be important to complement (and partially supersede) the currently scattered information (e.g., [2,4,8,12])

### 1.2. Primary DNA and protein sequence databases

### EMBL, GenBank and DDBJ

**http://www.ebi.ac.uk/embl**
**http://www.ncbi.nlm.nih.gov/Genbank**
**http://www.ddbj.nig.ac.jp/**

EMBL/GenBank/DDBJ is a collaboration of the primary nucleotide sequence databases. *S. pombe* genome project data and updates are submitted directly to EMBL. The three databases are synchronised on a daily basis, and the accession numbers are managed consistently. These databases are redundant and provide minimal error checking.

### TrEMBL

**http://www.ebi.ac.uk/swissprot**

TrEMBL contains the automatically annotated translations of known and predicted coding sequences (CDS) present in the EMBL database that are not yet integrated into SWISS-PROT and can be considered as a preliminary section of SWISS-PROT. Entries are assigned SWISS-PROT accession numbers (e.g., P04551) but no identifiers (e.g., CDC2_SCHPO).

### 1.3. Curated protein and protein domain databases

### SWISS-PROT

**http://www.ebi.ac.uk/swissprot**

SWISS-PROT consists of curated, non-redundant sequence entries. It contains high-quality annotation and is cross-referenced to several other databases. A complete list of the *S. pombe* entries curated into SWISS-PROT is accessible at: **http://expasy.ch/cgi-bin/lists?pombe.txt**. SWISS-PROT release 40.0 contains 1842 curated *S. pombe* entries; the remaining 3672 entries are in TrEMBL and will

Copyright © 2002 John Wiley & Sons, Ltd.

be curated into SWISS-PROT with the removal of redundant entries.

#### PombePD

**http://www.incyte.com/sequence/proteome/databases/PombePD.shtml**

PombePD is a commercial database developed by Proteome Inc. with much initial input from the fission yeast community [7]. It is now part of the BioKnowledge® library of Incyte Genomics. Despite previous promises to contributors [7], Incyte has recently started to charge yearly subscription fees, even for academic users. PombePD provides curated reports for each *S. pombe* protein and is integrated with databases of other organisms within the library. Weekly updates add new scientific content from the literature. In April 2002, 989 fission yeast proteins were listed as characterized by genetics or biochemistry, as reported in 2451 references.

#### InterPro

**http://www.ebi.ac.uk/interpro/index.html**

Protein sequence signature databases such as PROSITE, PRINTS, SMART, Pfam, ProDom, and TIGRFAMs are vital resources for identifying potential motifs and domains, particularly in novel sequences. InterPro (URL above) is a collaboration between these databases and provides an integrated resource of defined signatures and a facility for text and sequence-based searches [1]. In addition, all of the participating databases provide sequence search options from their individual websites (Pfam and TIGRFAMs also allow the adjustment of thresholds to enable the identification of less conserved domains). Protein signature searches were an integral part of the primary fission yeast annotation and are increasingly important as a resource for 'domain-driven' researchers.

### 1.4. Gene Ontology Consortium

#### GO

**http://www.geneontology.org/**

The Gene Ontology (GO) Consortium provides 'a dynamic controlled vocabulary that can be applied to any organism even as knowledge of gene and protein roles in cells is accumulating and changing' [5,6]. A common vocabulary to describe the attributes of gene products will facilitate consistent comparisons between organisms and will allow the automated querying of genes and proteins based on shared biology. It will also aid the interpretation of large datasets created by functional genomics projects [6]. The majority of eukaryotic genome projects already use the GO annotation system, and GO annotations are being incorporated into SWISS-PROT and GeneDB (see section 1.5).

Gene products are annotated using three GO ontologies: biological process, molecular function, and cellular component. Each ontology contains a set of well-defined terms with clearly described, specific relationships to each other. To represent biological reality accurately, the GO vocabularies are structured such that any term may have multiple parents as well as zero, one, or more children. A gene product may be annotated to a term at any level within the ontology. Because annotation to a term implies assigning its parents, a gene product can be retrieved from a search for the actual terms assigned to it, or for parent terms.

GO is continually expanded and altered to reflect increasing biological knowledge. To facilitate this process, suggestions for new terms, or alterations to existing ontologies can be submitted via the GO website above. The ontologies can be searched and browsed using a number of specially designed tools such as the AmiGO ontology browser at **http://www.godatabase.org/cgi-bin/go.cgi**. This tool also allows access to all gene products annotated to specific terms from all the participating databases. Assignments to GO terms are attributed to a source, which may be a published paper, a database cross-reference, or a computational analysis, and indicate the type of evidence supporting the annotation. Evidence types include 'inferred from mutant phenotype' (abbreviated IMP), 'inferred from direct assay' (IDA) and others.

### 1.5. Fission yeast genome database

#### GeneDB

**http://www.genedb.org/pombe**

**Database development.** Fission yeast is one of the initial organisms funded for inclusion into the GeneDB genomics database being developed at the Wellcome Trust Sanger Institute. The GeneDB project will develop and maintain database resources to support sequence and annotation at both the DNA and protein level. It will also

Copyright © 2002 John Wiley & Sons, Ltd.

provide a repository for the storage of data derived from functional genomics projects (see section 3). Integration of various data with existing information will help to interpret data within the framework of the whole genome. Functionality for the annotation and curation of features and attributes of both DNA (e.g., genes, transcripts, exons, introns, UTRs, promoters, repeats) and proteins (e.g., functions, domains, interactions, phenotype) will be provided. The resource will also display the results of predictive software (e.g., signal sequences, transmembrane helices, domains). Sequence visualisation will be provided initially by map and contig views, and in the longer term by additional views (e.g., interaction, pathway). Extensive cross-references will allow retrieval of related information from external resources. Search tools, comprehensive data retrieval facilities, and a helpdesk will provide levels of access suitable for both novice and expert users.

A prototype of GeneDB is now available which includes one-page reports for each protein-coding gene. These pages provide basic information, location details, predicted peptide properties, GO associations, domain information, database cross references, and sequence access. A BLAST server and browseable catalogues of annotated descriptions, GO associations, and Pfam domains are also available.

**Fission yeast curation within GeneDB.** Fission yeast annotations are updated on a daily basis to reflect new characterizations from EMBL/GenBank submissions, publications, and user feedback. The annotation currently provides basic descriptive infor mation including known or predicted compartment, process and function, presence of domains, and similarity to budding yeast (closest homolog). At present, 3443 genes have some functional information attached, ~1300 from published data, and the remainder inferred from similarity.

Annotations have been manually curated to include domain descriptions using Pfam (see section 1.3; [3]). Pfam provides high coverage for fission yeast (more than 65%, which is higher than any other eukaryote), with a low incidence of false positives. Domain identification is also an ongoing process, and new domains are continually identified and included in the core annotation.

GO associations (see section 1.4) for *S. pombe* genes are currently created semi-automatically, by comparing the curated annotations to a set of curated keywords that are always associated with a particular GO term. As an example, Figure 2 shows a list of the terms from the 'cellular component' ontology to which the *S. pombe* Arp2/3 complex proteins have been assigned. All seven identified fission yeast Arp2/3 complex proteins are annotated as 'Arp2/3 actin-organizing complex', and this structured syntax is used to assign these genes to the 'Arp2/3 protein complex' term and its 'parent' terms shown in Figure 2. Similarly, these proteins are assigned to several GO terms under 'biological process', the most specific one being 'actin cytoskeleton organization and biogenesis'. More specific 'child' terms are available, including 'actin nucleation' and 'actin filament organization'. Fission yeast genes have not yet been assigned to these terms, so the higher-level category serves as a 'place holder' for later refinement of the associations. New fission yeast annotations use structured syntax wherever possible; this not only enables preliminary GO assignments to be automated, but also allows similar annotations to be grouped together and browsed in GeneDB.

- GO:0005623 cell

  - GO:0005622 intracellular

    - GO:0005737 cytoplasm

      - GO:0005856 cytoskeleton

        - GO:0015629 actin cytoskeleton

          - GO:0005885 Arp2/3 protein complex

**Figure 2.** GO terms of the 'cellular component' category to which the Arp2/3 complex proteins of fission yeast have been assigned

For fission yeast genes, the annotations currently use only 130 of the 4747 available 'biological process' terms (9221 assignments), and 82 of the 5010 available 'cellular component' terms (4207 assignments), but many terms are not relevant to yeast. The next phase of the fission yeast annotation will involve the manual curation of GO assignments, with the addition of evidence codes and supporting citations (see section 1.4). No fission yeast genes have, as yet, been assigned to the 'molecular function' ontology, but this is also planned for the future. The long-term aim for fission yeast (as for other organisms: [10]) is to associate each characterised gene product with one or more GO terms.

Copyright © 2002 John Wiley & Sons, Ltd.

### 2. Data submissions, updates, and user feedback

Databases rely heavily on the research community to maintain correct and up-to-date information. User submissions and feedback are important for correcting gene prediction in addition to maintaining up-to-date annotation. Public databases would become immediately more reliable if every expert provided updates to the database entries for their favourite genes. Relatively small efforts by individual researchers could have dramatic impacts on the usefulness of genomic databases. This is particularly important for fission yeast considering its relatively small community and limited resources. Below, we describe how users can submit and update data in the various databases.

### EMBL Webin, GenBank BankIt and DDJB

**http://www.ebi.ac.uk/embl/Submission/webin.html**
**http://www.ncbi.nlm.nih.gov/BankIt/**
**http://www.ddbj.nig.ac.jp/updt-e.html**
To submit to EMBL/GenBank/DDBJ use the forms and guidance provided at the URLs above. Individual submissions can be made to any of these collaborating databases. It is important that users maintain their original EMBL/GenBank/DDBJ submissions as these cannot be altered by third parties. Original entries should be updated to correct sequencing errors, update citations, and add features by making use of available feature qualifiers such as intron, promoter, or polyA signal (see **http://www3.ebi.ac.uk/Services/WebFeat/**for a complete list). This will allow experimental nucleotide data to be transferred onto the genome sequence in GeneDB (see section 1.5) where they will be available non-redundantly and ultimately searchable in the context of the genome. As the primary nucleotide databases are redundant, re-sequenced regions can also be submitted as new entries with additional features.

### SWISS-PROT updates

**http://www.expasy.org/sprot/sp_update_form.html**
Here you can submit corrections or updates (published or unpublished) to the *S. pombe* SWISS-PROT curator.

### Gene Registry

**http://www.genedb.org/genedb/pombe/GeneRegistry.jsp**
A Gene Naming Committee lead by Takashi Toda has been set up to coordinate gene names in fission yeast and reserve names ahead of publication. The committee aims to resolve existing gene name conflicts (e.g., identical name for different genes, non-standard nomenclature, or different names for same gene). In addition, it is hoped that consistent names can be implemented wherever possible for newly defined genes. The committee can be contacted at the URL above or by e-mailing GNC@sanger.ac.uk. New gene designations will be circulated to the community via pombelist, a fission yeast mailing list (see **http://www.sanger.ac.uk/Projects/S_pombe/pombe_list.shtml**for subscription information). Once accepted, the new information will be forwarded to the other public databases.

### GeneDB

**http://www.genedb.org/genedb/pombe/curator.jsp**
Updates can be submitted to GeneDB through the general update form at the URL above, or using the forms provided on the individual gene pages. Submission forms will be structured to simplify the submission of experimental data and supporting publications. Additional data, comments, and suggestions outside the scope of the submission forms can be submitted directly to the curator or the database developers and are actively encouraged. Functional genomics data will also be incorporated, and submitters should contact the curator to discuss submission formats and data types to ensure rapid inclusion in GeneDB.

### 3. Resources for functional genomics

To increase accessibility and comparison of post-genomic datasets within and between organisms, it will be important to develop central data resources similar to public sequence databases. Post-genomic data are typically much more complex than sequence data, but promising initiatives have been launched to set standards for recording and reporting microarray-based gene expression experiments [4]. For budding yeast, user-friendly resources to visualize and survey microarray and other functional genomics data have been established [2], [8],

Copyright © 2002 John Wiley & Sons, Ltd. 

[12]. The fission yeast post-genomic era has only just started. Resources are therefore still limited and will probably change and develop rapidly over the coming years. Below, we list some sites that provide functional genomic information and tools:

1. **http://pombe.biols.susx.ac.uk/**

FYSSION: Strain database (published strains, temperature-sensitive library, and insertional mutants) maintained by the Armstrong group (University of Sussex).

2. **http://www-karc.crl.go.jp/bio/GFP-lib/htmls1/sum.html**

Protein localization: Searchable library of localization patterns, based on large-scale screening of a GFP-fusion library by the Hiraoka group [9].

3. **http://www.sanger.ac.uk/PostGenomics/S_pombe**

DNA microarrays: Protocols, project information, and data on genome-wide expression profiling and other functional genomics studies from the Bähler group (Sanger Institute) and collaborators.

### 4. General information, protocols, and tools

1. **http://pingu.salk.edu/~forsburg/lab.html**

Information on fission yeast biology, protocols, plasmids and other resources such as a community newsletter; maintained by the Forsburg group (Salk Institute).

2. **http://www.bio.uva.nl/pombe/handbook**

Fission yeast handbook: A protocol collection from the Nurse group; hosted by F. Hochstenbach (University of Amsterdam).

3. **http://megasun.bch.umontreal.ca/People/lang/species/spo/spombe.html**

FMGP: Mitochondrial genome data provided by F. Lang (Montreal University).

4. **http://www.cbs.dtu.dk/services/GenomeAtlas**

GenomeAtlas at CBS (Technical University of Denmark): Structural chromosome maps to visualize various features and architecturally important regions within large regions of DNA.

5. **http://www.sanger.ac.uk/Software/Artemis/**

Artemis is a DNA sequence viewer and annotation tool developed at the Sanger Institute that allows visualization of sequence features and analysis results within the context of the sequence and its six-frame translation [13]. It is written in Java and available for UNIX, GNU/Linux, BSD, Macintosh, and MS Windows operating systems. In addition to supporting the annotation effort of *S. pombe* and other organisms, this tool is increasingly popular on the desktops of experimental biologists. It can be used to browse and search sequence contigs available on the *S. pombe* ftp site (**http://www.sanger.ac.uk/Projects/S_pombe/ftp.shtml**). Some useful features of Artemis include:

- Display plots of sequence composition (e.g., GC content) alongside the sequence
- Create personal annotations by adding new features (e.g., restriction sites)
- Search for DNA or protein sequence patterns, keyword text, or various features (e.g., CDS, tRNA, UTR)
- Export in different formats (e.g., sequence lists or regions)
- Plots of protein features (e.g., hydrophobicity, hydrophilicity, coiled-coil)

A comprehensive user manual describing all the available features of Artemis is available at the URL above.

### Acknowledgement

We thank the many colleagues of the fission yeast community for their feedback and updates that are so crucial for annotation and curation, Midori Harris for help with the GO database section, and Al Ivens for reading of the manuscript. VW and JB are supported by the Wellcome Trust and Cancer Research UK, respectively.

### References

1. Apweiler R, Attwood TK, Bairoch A, *et al.* 2001. The InterPro database, an integrated documentation resource for protein families, domains and functional sites. *Nucleic Acids Res* **29**: 37–40.
2. Ball CA, Jin H, Sherlock G, *et al.* 2001. *Saccharomyces* genome database provides tools to survey gene expression and functional analysis data. *Nucleic Acids Res* **29**: 80–81.
3. Bateman A, Birney E, Cerruti L, *et al.* 2002. The Pfam protein families database. *Nucleic Acids Res* **30**: 276–280.
4. Brazma A, Hingamp P, Quackenbush J, *et al.* 2001. Minimum information about a microarray experiment (MIAME)-toward standards for microarray data. *Nature Genet* **29**: 365–371.
5. The Gene Ontology Consortium. 2001. Creating the gene ontology resource: design and implementation. *Genome Res* **11**: 1425–1433.
6. Ashburner M, Ball CA, Blake JA, *et al.* 2000. Gene Ontology: tool for the unification of biology. *Nature Genet* **25**: 25–29.
7. Costanzo MC, Crawford ME, Hirschman JE, *et al.* 2001. YPD, PombePD and WormPD: model organism volumes of

Copyright © 2002 John Wiley & Sons, Ltd. 

the BioKnowledge library, an integrated resource for protein information. *Nucleic Acids Res* **29**: 75–79.

8. Crom SL, Devaux F, Jacq C, Marc P. 2002. yMGV: helping biologists with yeast microarray data mining. *Nucleic Acids Res* **30**: 76–79.
9. Ding D-Q, Tomita Y, Yamamoto A, Chikashige Y, Haraguchi T, Hiraoka Y. 2000. Large-scale screening of intracellular protein localization in living fission yeast cells by the use of a GFP-fusion genomic DNA library. *Genes to Cells* **5**: 169–190.
10. Dwight SS, Harris MA, Dolinski K, *et al*. 2002. *Saccharomyces* Genome Database (SGD) provides secondary annotation using the Gene Ontology (GO). *Nucleic Acids Res* **30**: 69–72.
11. Eisen JA. 2002. Brouhaha over the other yeast. *Nature* **415**: 845–848.
12. Marc P, Devaux F, Jacq C. 2001. yMGV: a database for visualization and data mining of published genome-wide yeast expression data. *Nucleic Acids Res* **29**: 63.
13. Rutherford K, Parkhill J, Crook J, Horsnell T, Rice P, Rajandream M-A, Barrell B. 2000. Artemis: sequence visualization and annotation. *Bioinformatics* **16**: 944–945.
14. Wixon J. 2002. Featured organism: *Schizosaccharomyces pombe*, the fission yeast. *Comp Funct Genom* **3**: 194–204.
15. Wood V, Gwilliam R, Rajandream M-A, *et al*. 2002. The genome sequence of *Schizosaccharomyces pombe*. *Nature* **415**: 871–880.
16. Yanagida M. 2002. The model unicellular eukaryote, *Schizosaccharomyces pombe*. *Genome Biol* **3**: comment 2003.1–2003.4.

Copyright © 2002 John Wiley & Sons, Ltd. 
